# Highly expressed STAT1 contributes to the suppression of stemness properties in human paclitaxel-resistant ovarian cancer cells

**DOI:** 10.18632/aging.103317

**Published:** 2020-06-09

**Authors:** Fanchen Wang, Lingyun Zhang, Jiao Liu, Jinguo Zhang, Guoxiong Xu

**Affiliations:** 1 Research Center for Clinical Medicine, Jinshan Hospital, Fudan University, Shanghai 201508, China; 2 Department of Oncology, Shanghai Medical College, Fudan University, Shanghai 200032, China; 3 Center for Tumor Diagnosis and Therapy, Jinshan Hospital, Fudan University, Shanghai 201508, China

**Keywords:** chemoresistance, methylation, ovarian cancer, STAT1, stem cell

## Abstract

Signal transducer and activator of transcription-1 (STAT1) is an important factor in various cellular processes. The cancer stem cell (CSC) is considered as a tumor-initiating cell that drives the inner hierarchy in many cancers including epithelial ovarian cancer (EOC). Here, we explored for the first time the regulation of STAT1 on stemness properties in chemoresistant EOC cells. The paclitaxel (PTX)-resistant EOC cell line (OV3R-PTX) was derived from PTX-sensitive OVCAR-3 cells treated by the PTX regimen. A single cell clone OV3R-PTX-B4 was selected by fluorescence-activated cell sorting. PTX-resistant cells grew slowly in conventional 2D and 3D cultures, but tumor xenograft with PTX-resistant cells grew fast in nude mice. Interestingly, OV3R-PTX-B4 cells shared the characteristics of CSCs and stemness properties were found to be increased in the non-adherent spheroid culture system. The PTX-resistant cells had a high expression of CSC-related markers and low expression of STAT1 that had a high methylation level of CpG in its promoter region. Overexpressed STAT1 suppressed stemness properties, cell proliferation, and colony formation and favored the overall survival of patients with EOC. In summary, these data indicate a regulatory mechanism of STAT1 underlying drug resistance and provide a potential therapeutic application for EOC patients with PTX resistance.

## INTRODUCTION

Epithelial ovarian cancer (EOC) is the most common ovarian cancer (OC) accounting for more than 90% of all cases and the age distribution shows that the highest incidence occurs at age 75-85 [1]. EOC is a highly lethal malignant disease. The five-year survival rate of patients with EOC is low because the disease is diagnosed too late, due to the lack of early detection, and relapse [2, 3]. The standard first-line treatment of EOC is debulking surgery followed by platinum combined with paclitaxel (PTX) chemotherapy. These therapies are often effective initially, but patients later face recurrence due in part to the development of multi-drug resistance, which contributes to poor prognosis [4, 5]. The accumulated evidence shows that cancer stem cells (CSCs), also known as stem cell-like cancer cells, play an important role during the processes of drug resistance and metastasis [6, 7]. A small subset of CSCs is deemed to be the real driving force of tumor development [8] and to share some common features with normal stem cells as they have the self-renewal capacity and proliferation potential [9, 10]. CSCs are often observed in tumors of patients with relapse and drug resistance, which have a self-renew property and are able to differentiate into heterogeneous lineages of cancer cells [6]. The chemoresistance arisen in recurrent EOC may be a result of the stem cell transformation, which drivers cancer cell heterogeneity [11].

Signal transducer and activator of transcription-1 (STAT1), one of the members of the STAT family, is involved in tumorigenesis [12]. As a signal transducer, transcription factor, and immune modulator [13], STAT1 plays an essential role in response to interferon (IFN) signaling and regulates many cellular processes, such as proliferation, differentiation, and cell death [14, 15]. High level of STAT1 is associated with improved chemotherapy response in high-grade serous OC, suggesting that STAT1 may be a biomarker of chemosensitivity [16]. Our previous study demonstrated the overexpression of STAT1 in high-grade serous OC and unveiled crosstalk between STAT1 and TGF-β signaling pathways in OC cells [17]. It has been shown that the TGF-β signaling pathway is one of the primary signaling pathways responsible for maintaining pluripotency and self-renewal abilities in embryonic and somatic stem cells [18, 19]. However, the regulation and effect of STAT1 on stemness properties in chemoresistant EOC cells are largely unknown.

In this study, a PTX-resistant cell line and a stem cell-like cell clone were generated. The CSC model was used to identify the effectiveness of STAT1 in EOC cells and to discover the regulatory mechanism of STAT1 on stemness properties in chemoresistant EOC cells. The DNA methylation status of STAT1 was also examined.

## RESULTS

### OV3R-PTX cells are highly resistant to paclitaxel

The OV3R-PTX cell line was derived from the parental cell line OVCAR-3 through multi-month passages by incrementally increasing doses of PTX. The IC_50_ of OV3R-PTX cells was 1.469 μM that was 100 times higher than its parental OVCAR-3 cells whose IC_50_ was 0.014 μM. Indeed, the DRI of OV3R-PTX was 104.93 after initial establishment and was 95.25 and 101.27 after frozen and thaw 1 month and 6 months, respectively, suggesting that the resistant phenotype is relatively stable. Comparing to OVCAR-3 cells that were dying after PTX treatment, OV3R-PTX cells grew well in the presence of 0.1 μM PTX for 48 h and even were survival after a high dose of PTX treatment (1 μM) in a conventional monolayer 2D culture (Supplementary Figure 1).

### PTX-resistant cells grow slowly compared to PTX-sensitive cells

**Figure 1:**
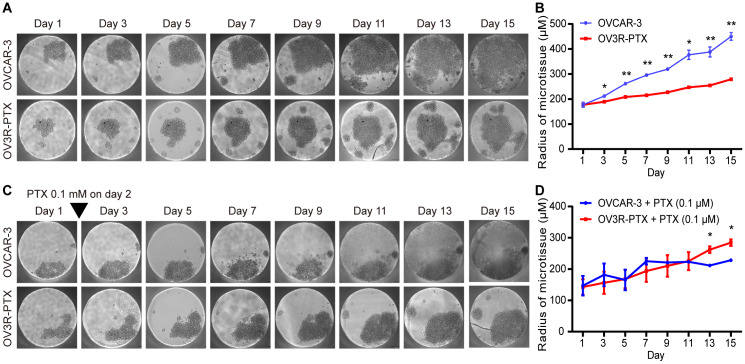
**Observation of micro-sphere in 3D cell culture.** OVCAR-3 and OV3R-PTX cells grew in 3D culture for 15 days. (**A**) Micro-sphere grew in 3D culture without PTX treatment. The pictures were taken by phase-contrast microscopy every 2 days. (**B**) Quantitative analysis of sphere diameter from A (n = 3 independent experiments). (**C**) Micro-sphere grew in 3D culture with PTX treatment. Cells were cultured in the presence of 0.1 μM PTX on day 2 and pictures were taken by phase-contrast microscopy every 2 days. (**D**) Quantitative analysis of sphere diameter from **C** (n = 3 independent experiments). Representative images are shown. An inverted triangle indicates one-shot treatment. Original magnification, × 100. *, *P* < 0.05; **, *P* < 0.01.

The impact of cell growth in monolayer culture was evaluated by doubling time which was found to be 20.40 ± 0.67 and 29.99 ± 1.58 h in OVCAR-3 and OV3R-PTX cells, respectively (Supplementary Figure 2), indicating a slow growth rate in PTX-resistant cells. Since the multicellular 3D culture model is the most commonly used in cancer research, next, both OVCAR-3 and OV3R-PTX cells were cultured in Gravity plates for 15 days. Similarly to 2D culture, OV3R-PTX cells grew slowly compared to OVCAR-3 cells in 3D culture (Figure 1A) and a micro-sphere of OV3R-PTX was found to be smaller than that of OVCAR-3 (Figure 1B). In the presence of 0.1 μM PTX, the growth of OVCAR-3 cells was suppressed (Figure 1C), whereas the growth of OV3R-PTX cells did not (Figure 1D), further demonstrating that OV3R-PTX cells resisted to PTX.

### Tumor with PTX-resistant cells grows fast in nude mice

**Figure 2:**
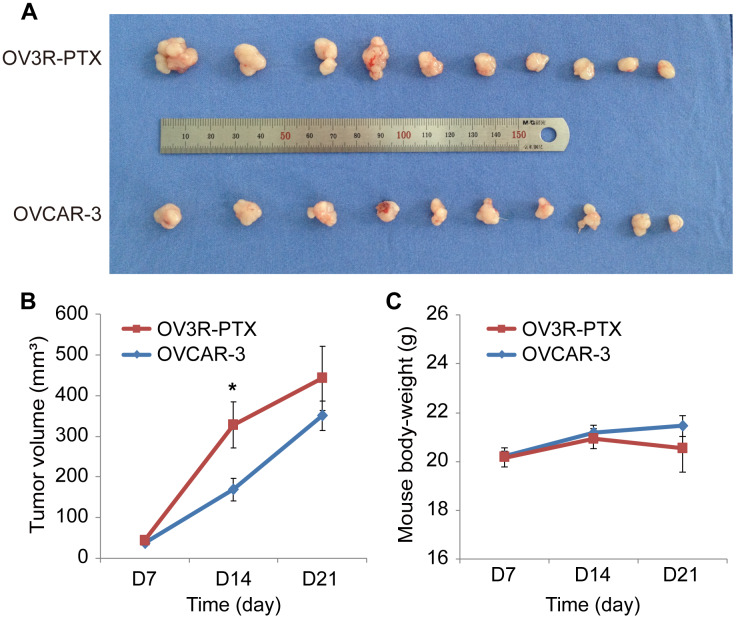
**Tumor growth in nude mice.** Mice were implanted with either OVCAR-3 cells or OV3R-PTX cells and lived for 3 weeks. (**A**) Picture of xenograft tumor mass after sacrifice. (**B**) Measurement of tumor volume in a time-course study. (**C**) Measurement of mouse body-weight in a time-course study. n = 10; *, *P* < 0.05.

Since OV3R-PTX cells grew slowly in 2D and 3D culture systems, next, we asked whether these cells grown *in vivo* would be similar to those *in vitro*. The tumor xenograft assay showed that tumors were formed in both OVCAR-3 and OV3R-PTX injected mice (Figure 2A). However, the tumor volume was different between the two groups after an equal amount of cells implanted into mice for 21 days. The volume of tumors was larger in OV3R-PTX mice than OVCAR-3 mice (Figure 2B), suggesting that OV3R-PTX cells may be more tumorigenic. However, the body-weight of mice was not different between the two groups (Figure 2C).

### Monoclonal PTX-resistant cells grow fast compared to PTX-sensitive cells

**Figure 3:**
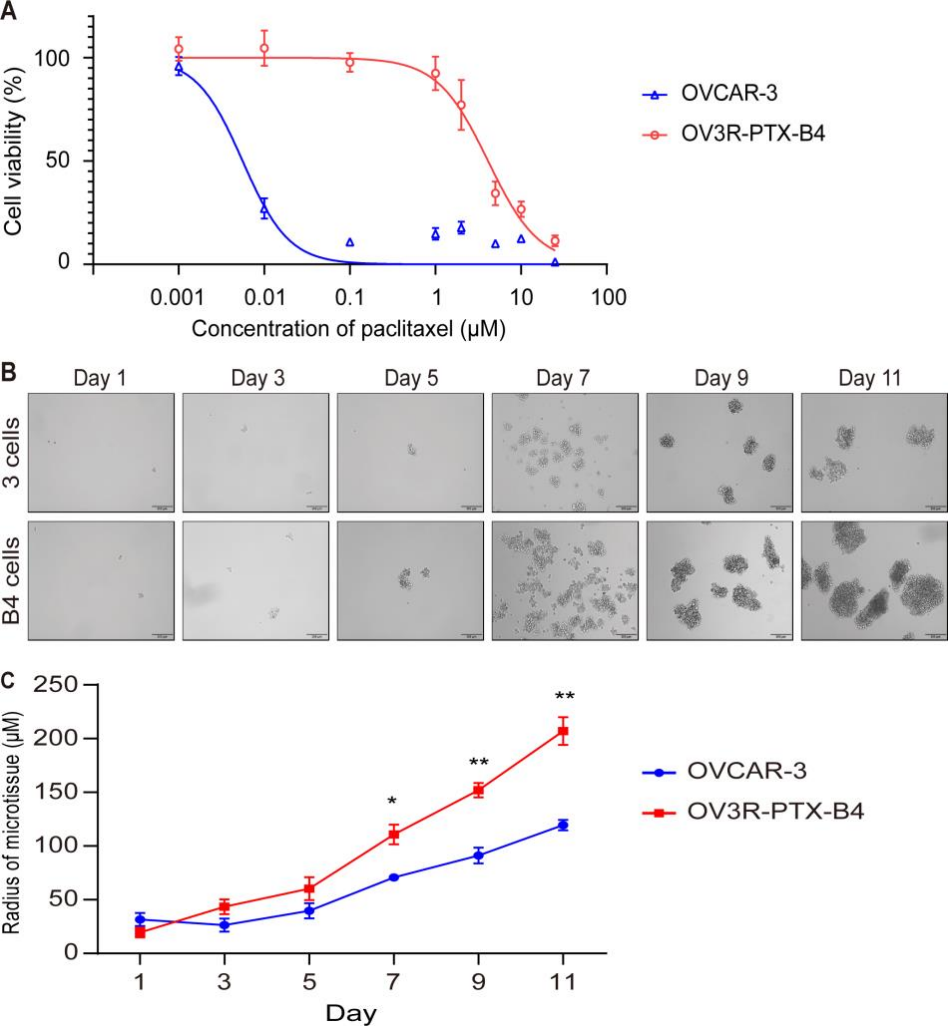
**Confirmation of monoclonal PTX-resistant cells.** (**A**) Cell viability curve. The viability of OVCAR-3 and OV3R-PTX-B4 cells that resisted to PTX were evaluated by the CCK-8 assay. OVCAR-3 and OV3R-PTX-B4 cells (4000 cells/well) were treated with PTX in a dose-dependent study (0.001 0.01, 0.1, 1, 2, 5, 10, and 25 μM/ml) for 48 h. (**B**) Capacity of spheroid formation. OVCAR-3 and OV3R-PTX-B4 cells were cultured in serum-free DMEM/F12 medium with EGF, bFGF, heparin, and B27 supplements under a low-adhesive condition for 11 days. The pictures were taken by phase-contrast microscopy every 2 days. Representative images are shown. (**C**) Quantitative analysis of spheroid diameter from **B**. n = 3 independent experiments; *, *P* < 0.05; **, *P* < 0.01.

Because OV3R-PTX cells grew slowly in 2D and 3D cultures but fast in tumor xenograft, we speculated that there is a mixture of heterogeneous cells in the OV3R-PTX cell population, in which stem cell-like cancer cells may exist. In order to obtain a subtype of resistant cells from OV3R-PTX, a single-cell clone that shares the characteristics of CSCs was selected using a FACS technique. A monoclonal cell line was isolated and developed, which was named OV3R-PTX-B4. This clone was confirmed to have a resistant phenotype by treating cells with PTX in a dose-dependent study (0.001 - 25 μM). The cell viability assay showed that OV3R-PTX-B4 had PTX-resistant properties compared with OVCAR-3 (Figure 3A). To further verify this difference, a spheroid formation assay was performed under a serum-free, low-adhesive CSC culture condition. OV3R-PTX-B4 had more ability to form a spheroid as a higher spheroid formation capacity was observed (Figure 3B, 3C). These results imply that tumors grown fast in vivo are most likely due to an outgrowth of stem cell-like cancer cells.

### OV3R-PTX-B4 cells share the characteristics of cancer stem cells

**Figure 4:**
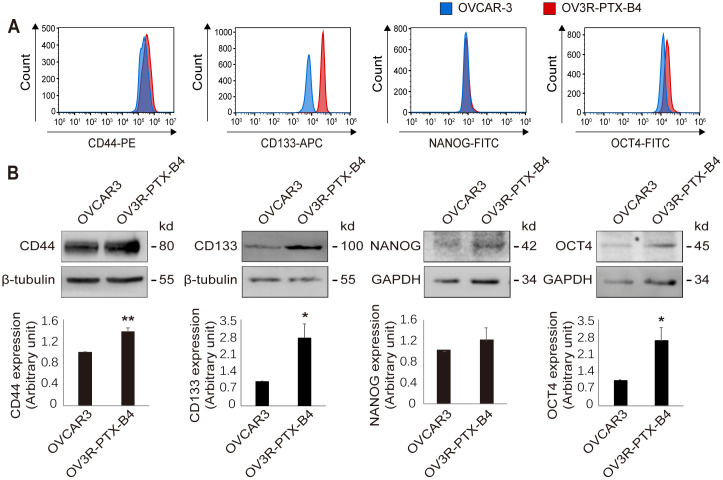
**Differential expression of stemness markers between OVCAR-3 and OV3R-PTX-B4 cells.** (**A**) Detection of CD44, CD133, NANOG, and OCT4 positive cell population in OVCAR-3 (blue) and OV3R-PTX-B4 cells (red) by flow cytometry. (**B**) Expression of CD44, CD133, NANOG, and OCT4 proteins in OVCAR-3 and OV3R-PTX-B4 cells detected by Western blot. Upper panel, representative images of blotting; low panel, semi-quantitative analysis of the relative optical density of protein bands in the upper panel. β-tubulin and GAPDH were used as loading controls. n = 3; *, *P* < 0.05; **, *P* < 0.01.

Using CSC marker labeling, subtypes of CD44, CD133, NANOG, and OCT4 positive cell population were examined in OVCAR-3 and OV3R-PTX-B4 cells by flow cytometry. The distribution of CD133 positive cells was observed to be different between OVCAR-3 and OV3R-PTX-B4 cells (Figure 4A). The expression levels of CD44, CD133, and OCT4 proteins were found to be significantly higher in OV3R-PTX-B4 cells than OVCAR-3 cells detected by Western blot (Figure 4B).

### Stemness of OV3R-PTX-B4 cells is enhanced in the spheroid culture system without serum

**Figure 5:**
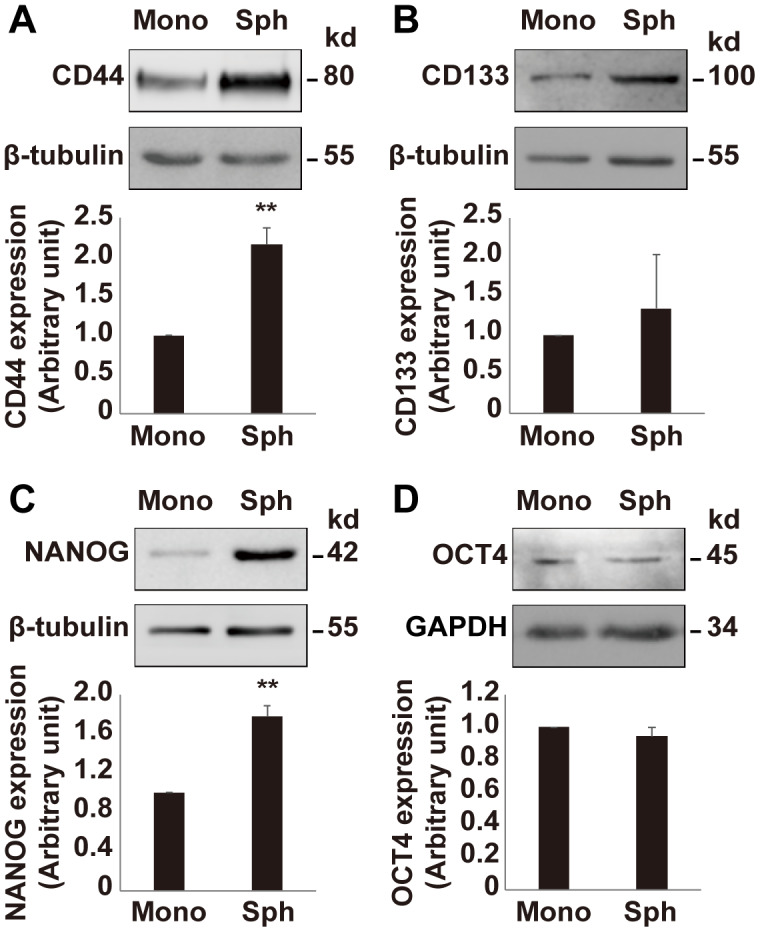
**Expression of stemness markers in OV3R-PTX-B4.** Cells were cultured under a monolayer culture system (Mono) or a spheroid culture system (Sph). The expression of CD44, CD133, NANOG, and OCT4 proteins were detected by Western blot. (**A**) CD44 expression. (**B**) CD133 expression. (**C**) NANOG expression. (**D**) OCT4 expression. Upper panel, representative images of blotting; low panel, semi-quantitative analysis of the relative optical density of protein bands in the upper panel. β-tubulin and GAPDH were used as loading controls. n = 3; **, *P* < 0.01.

Since the growth rate of OV3R-PTX-B4 cells is different between monolayer and spheroid cultures, next, we validated the expression of stemness-related markers in OV3R-PTX-B4 under these two different culture systems. The stemness-related markers CD44, CD133, NANOG, and OCT4 were detected by Western blot and found to be differentially expressed between these two culture systems. The expression of CD44 and NANOG proteins was higher in spheroid cells than monolayer cells (Figure 5A–5D), indicating that a spheroid culture system can maintain and enhance the stemness of PTX-resistant cells.

### STAT1 expression is decreased in PTX-resistant cells

Next, the expression of STAT1 between PTX-sensitive and -resistant cells and between monolayer and spheroid cells was examined. The expression of STAT1 mRNA was lower in PTX-resistant OV3R-PTX cells than PTX-sensitive OVCAR-3 cells detected by qRT-PCR (Supplementary Figure 3A). Western blot showed a similar result that STAT1 was downregulated in OV3R-PTX cells (Supplementary Figure 3B). The expression of STAT1 protein was significantly lower in OV3R-PTX cells than OVCAR-3 cells (*P* < 0.05; Supplementary Figure 3C).

### Hypermethylation of DNA in the promoter region leads to low expression of STAT1 in paclitaxel-resistant cells

**Figure 6:**
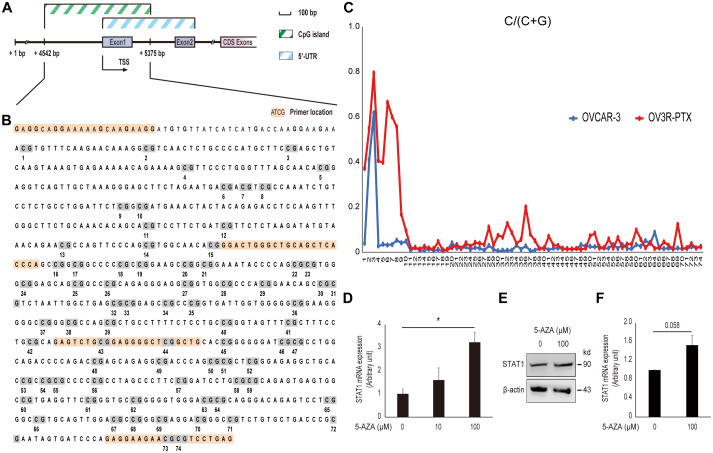
**Effect of DNA methylation on STAT1 expression.** (**A**) Schematic illustration of the STAT1 promoter region. The CpG island and 5’-untranslated region (UTR) are indicated. Numbers point the position of base pair (bp) in the sequence of GenBank (accession # NG_008294.1). CDS, coding sequence; TSS, transcription start site. (**B**) DNA sequence of CpG island in the STAT1 promoter region. The full-length CpG-rich promoter region was 834 bp in length. The primers used for MSP and DNA sequencing are highlighted in orange and CpG sites with numbers under the sequence are highlighted in grey. (**C**) Methylation level in a STAT1 promoter region detected by bisulfite sequencing. Each CpG site in the STAT1 promoter region was labeled on the horizontal axis (74 sites in total). The scale in the vertical axis represented the C to C+T ratio, which reflected the methylation level of the relevant CpG site. (**D**) STAT1 mRNA expression. OV3R-PTX cells were treated with 0, 10, and 100 μM 5-AZA, respectively, for 72 h. DMSO was used as a control. The mRNA level was detected by qRT-PCR. n = 3; *, *P* < 0.05. (**E**) STAT1 protein expression detected by Western blot after 100 μM 5-AZA for 72 h. β-actin was used as a loading control. Representative images of blotting are shown. (**F**) Semi-quantitative analysis of the relative optical density of protein bands in E. n = 3; *, *P* = 0.058.

In order to address why STAT1expression was low in resistant OV3R-PTX cells, CpG islands in the STAT1 promoter region were analyzed using the UCSC Genome Browser database (http://genome.ucsc.edu/) and a schematic illustration was drawn (Figure 6A). About 74 CpG sites were found in the promoter region of STAT1 (Figure 6B). After bisulfite modification of DNA, the methylation status of STAT1 DNA in the promoter region was vilified by MSP and Sanger sequencing. Hypermethylation CpG sites were detected in OV3R-PTX cells, but less methylation in OVCAR-3 cells (Figure 6C). To further confirm the promoter methylation involved in the downregulation of STAT1, OV3R-PTX cells were treated with 5-AZA for 72 h. STAT1 expression was upregulated after 100 μM AZA treatment (Figure 6D–6F), indicating the expression of STAT1 was restored. These data suggest that the hypermethylation of DNA in the promoter region was at least in part the cause of low expression of STAT1.

### Knockdown of STAT1 enhances stemness in OV3R-PTX-B4 cell

Since we had observed a different rate of cell growth between monolayer and spheroid culture systems, next, we examined the expression of STAT1 in cells under these two conditions. The expression of STAT1 protein was lower in OV3R-PTX-B4 cells under the spheroid culture condition than the monolayer culture condition (Supplementary Figure 4A, 4B).

**Figure 7:**
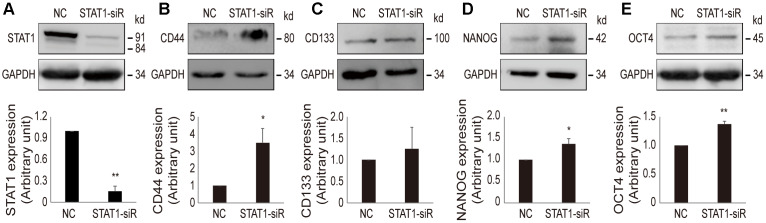
**Effect of STAT1-siRNA on stemness markers in OV3R-PTX-B4 cells.** Cells were transfected with STAT1-siRNA (STAT1-siR) or negative control (NC) scramble siRNA. The expression of STAT1, CD44, CD133, NANOG, and OCT4 proteins were detected by Western blot. (**A**) STAT1 expression. (**B**) CD44 expression. (**C**) CD133 expression. (**D**) NANOG expression. (**E**) OCT4 expression. Upper panel, representative images of blotting; low panel, semi-quantitative analysis of the relative optical density of protein bands in the upper panel. GAPDH was used as a loading control. n = 3; *, *P* < 0.05; **, *P* < 0.01.

In order to confirm that a low level of STAT1 can enhance stemness property in resistant cells, STAT1 was knocked down by STAT1-siRNA in OV3R-PTX-B4 cells (Figure 7A). The suppression of STAT1 significantly increased the protein expression of CD44, NANOG, and OCT4 but not CD133 (Figure 7B–7E). This observation further proved the negative regulation of STAT1 on stemness in PTX-resistant EOC cells.

### STAT1 is an inhibitory factor of stemness and influences OV3R-PTX-B4 cell behavior

**Figure 8:**
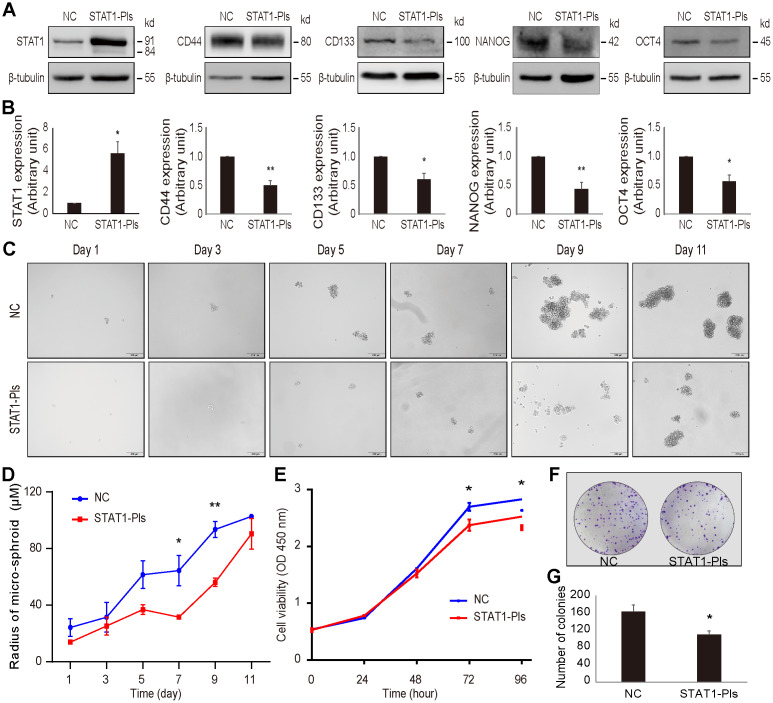
**Overexpression of STAT1 in OV3R-PTX-B4 cells.** Cells were transfected with STAT1 plasmid (STAT1-Pls) or negative control vector (NC). The overexpression of STAT1 was confirmed by Western blot. (**A**) The expression of STAT1, CD44, CD133, NANOG, and OCT4 in STAT1-overexpressing cells. Representative images of blotting are shown. β-tubulin was used as a loading control. (**B**) Semi-quantitative analysis of the relative optical density of protein bands in **A**. (**C**) Spheroid formation assay of the cells. Cells were cultured in serum-free DMEM/F12 medium with EGF, bFGF, heparin, and B27 supplements for 11 days. Photos were taken every two days. (**D**) Measurement of the diameter of each spheroid in **C**. (**E**) Cell viability measurement by the CCK-8 assay. (**F**) Colony formation. A representative picture of the colonies is shown. (**G**) Number of colonies is shown. n = 3 independent experiments; *, *P* < 0.05; **, *P* < 0.01.

Using gain-of-function approaches, we further examined the effect of STAT1 on stemness-related markers in OV3R-PTX-B4 cells. The expression of CD44, CD133, NANOG, and OCT4 proteins in OV3R-PTX-B4 cells was significantly suppressed by the overexpression of STAT1 after STAT1 plasmid transfection (Figure 8A, 8B).

Next, we examined the effect of STAT1 on spheroid formation under a serum-free, low-adhesive CSC culture condition. Overexpression of STAT1 reduced the OV3R-PTX-B4 cell spheroid formation (Figure 8C, 8D). Moreover, the overexpression of STAT1 significantly decreased OV3R-PTX-B4 cell viability (Figure 8E) and colony formation (Figure 8F). The number of colonies was less in the STAT1-overexpressing group (Figure 8G).

### High expression of STAT1 may be a protective factor in ovarian cancer

**Figure 9:**
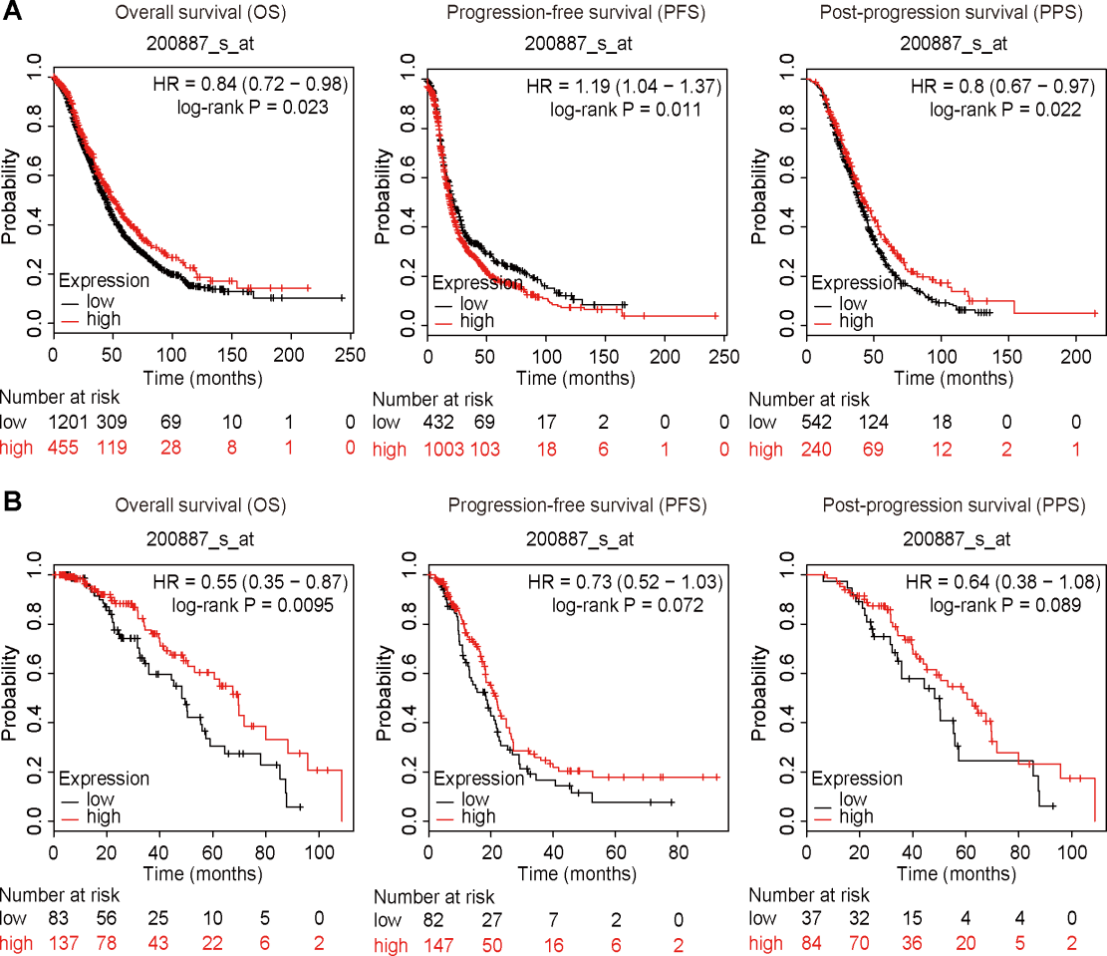
**Survival plots.** All patients were divided into two groups based on the expression level of STAT1: a high expression group and a low expression group. Data were extracted from a microarray (Affymetrix ID: 200887_s_at). The hazard ratio (HR) with 95% confidence intervals (in parentheses) and log-rank P for OS, PFS, and PPF were calculated, respectively. (**A**) Kaplan-Meier curves of the OS, PFS, and PPS of all patients with OC are presented. (**B**) Kaplan-Meier curves of the OS, PFS, and PPS of PTX-treated patients with OC are presented. Number, case number; OV, ovarian cancer; low, low expression of STAT1; high, high expression of STAT1; PTX, paclitaxel; STAT1, signal transducer and activator of transcription-1.

The survival data were extracted and calculated using the Kaplan-Meier plotter (https://kmplot.com/analysis/). High expression of STAT1 was positively correlated with OS and PPS but negatively correlated with PFS of all OC patients (Figure 9A). Furthermore, high expression of STAT1 influenced OS of PTX-treated patients with OC (Figure 9B), suggesting that a high level of STAT1 expression may favor the OS of patients with OC.

## DISCUSSION

Malignant tumors consist of a mixed cell population in which CSCs represent a small number of tumor cells in the subpopulation that are often observed and related to metastasis, relapse, and chemoresistance. The current study demonstrated for the first time that a general population of PTX-resistant EOC cells had heterogeneous lineages and a single-cell clone OV3R-PTX-B4 contributes toward a stemness phenotype which can be used as a stem cell model for the study of the mechanisms of PTX-resistance in EOC.

Tumor development and cancer cell fate mostly depend on the microenvironment. The current study using different culture systems showed different cell growth phenomena. Interestingly, we found that the growth rate was quite different between monolayer and spheroid cultures when we used the same cell line. Under a conventional culture condition, PTX-resistant cells grew slower compared to PTX-sensitive cells. However, in nude mice and cells cultured under a stem cell culture condition, the results were opposite which cells grew fast. These outcomes prompted us to think about two issues, the growth microenvironment and the stem cell-like cancer cells in a mixed cell population. It has been shown that most tumors possess the phenotypic heterogeneity which was found in the original tumor cell population [20]. In general, the tumors reside in and interact with their microenvironment [21]. The surface molecules can mediate interactions between cells and tumor microenvironment. The change of the microenvironment such as culture condition *in vitro* or stem cell niche *in vivo* may lead to the alteration of cell phenotypes [22, 23]. For example, some differentiated breast cancer cell lines cultured *in vitro* can convert to CSCs [24]. However, how cancer cells acquire stem cell capacity is not completely understood.

Accumulated studies suggest that chemoresistance is strongly linked with CSCs that reside in the tumor and show stem cell-like properties [25, 26]. The primary impetus of this study was to generate a cell line that is highly resistant to PTX. We purified and propagated cells when a stem cell-like cancer cell was selected. Indeed, a single-cell clone OV3R-PTX-B4 derived from the pool of drug-resistant cells had the properties of stem cells containing the stemness-related markers such as CD44, CD133, NANOG, and OCT4 which are generally used for identifying CSCs [27]. CD44 and CD133 are cell-surface proteins [28, 29], whereas NANOG and OCT4 are transcription factors [30]. All these four markers have been reported to be expressed in ovarian cancer [31–34].

Our recent studies show that STAT1 and TGF-β affect ovarian malignant tumor growth, progression, and metastasis [17, 35, 36]. The present study observed a low expression level of STAT1 in PTX-resistant cells, which was due to the fact that DNA in the promoter region of STAT1 was hypermethylated, compared with PTX-sensitive cells. It has been reported that the JAK/STAT signaling pathway plays an important role in the regulation of stem cells [37]. Using gain- and loss-of-function approaches, we unveiled a mechanism underlying the negative regulation of STAT1 on stemness-related markers in EOC cells.

Recent studies indicated that the relationship between CSCs and non-CSCs is not supposed to be entirely static, suggesting some cancer cells may exhibit plasticity and transition between stem and non-stem state [8, 38]. The change of STAT1 expression level may influence EOC cell stemness. Our data verified that the overexpression of STAT1 in stem cell-like PTX-resistant OC cells resulted in a decrease of stemness, inhibition of growth, and reduction of colony formation *in vitro*. It has been reported that STAT1 is involved in biological behaviors of cancers [39, 40], remodeling of the tumor microenvironment [41], and stemness properties [42]. The inhibitory effect of STAT1, to a certain extent, is related to its stemness and thus targeting CSCs based on their stem cell-related markers becomes possible in therapeutic applications [43].

Kaplan-Meier plotter analyses showed that the high expression of STAT1 had a good prognosis compared to the low or negative expression of STAT1, suggesting that STAT1 may be a protective factor in OC. Considering cancer stem cells are deemed to drive the progression and relapse of cancer, these data may support the perspective that STAT1 is a inhibit factor of stemness. By contrast, STAT1 showed to be a negative correlation with PFS, which suggests that the role of STAT1 in patients with EOC is complex. In patients with PTX treatment, overexpression of STAT1was also in favor of the OS of patients with EOC. Previous studies have indicated that STAT1 is an inhibitory factor in the development of several cancers [44] and highly expressed STAT1 is associated with longer OS of patients with OC [45].

Finally, we must point that a few limitations may exist when we used cancer cell lines and their derivatives across this study. The results of the study of stem cell biology and drug-resistance *in vitro* might differ that *in vivo* in cancer patients or animals. Cancer cells can acquire resistance to chemotherapy by a range of mechanisms. We believe that only a small fraction of cells possess stem cell characteristics but how a drug-resistance is triggered by these cells remains unclear. Why the DNA of STAT1 is hypermethylated in PTX-resistant cells? How heavy the factor of STAT1 weigh in patients who relapse because of chemoresistance? Does our cell model fit in the content of the clinical concern of drug resistance? These problems need to be solved in the future.

**Figure 10:**
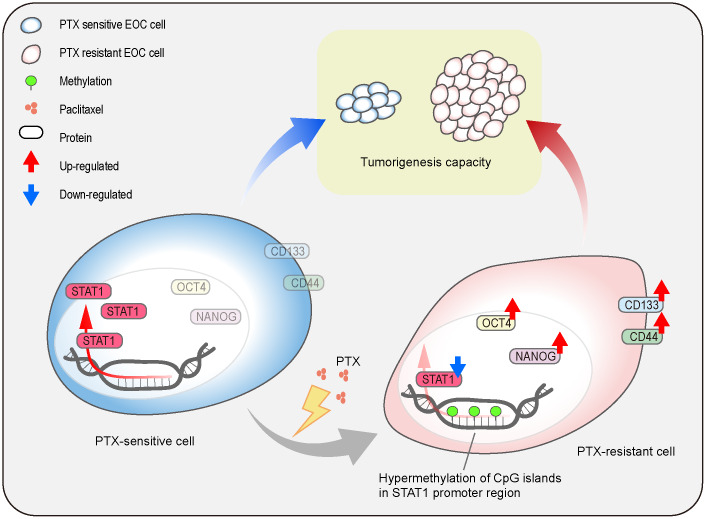
**Schematic model.** The illustration indicates the regulatory mechanism of STAT1 on stemness properties, which affect tumorigenesis capacity, in PTX-sensitive and PTX-resistant cells.

In summary, the current study shows the generation of a PTX-resistant cell model with stem-like properties and reveals the regulatory mechanism of STAT1 on stemness properties in PTX-resistant cells (Figure 10). Repeated treatment of PTX can induce EOC cell chemoresistance which causes the downregulation of STAT1 and enhances stem cell-related markers. The low expression of STAT1 in PTX-resistant cells is due to the hypermethylation of the CpG islands in its promoter region. Overexpression of STAT1 results in the suppression of EOC stemness and decreases tumorigenesis capacity. Since CSCs are the driving power of cancer development, recurrent, and chemoresistance and STAT1 influences the stemness of CSCs, a novel therapeutic strategy can be made by targeting STAT1 for the treatment of patients with PTX-resistance.

## MATERIALS AND METHODS

### Monolayer (2D) and three-dimensional (3D) cell culture

All of these cells were cultured in RPMI-1640 medium (Sigma-Aldrich, St. Louis, MO, USA) supplemented with 10% fetal bovine serum (FBS, Gibco-Invitrogen, Grand Island, NY, USA) (complete medium) and maintained in monolayer (two-dimensional, 2D-culture) in Petri dish or flask unless otherwise indicated.

The three-dimensional (3D) growing assay with spherical structure and uniform size was performed in a 96-well plate using a GravityPLUS^TM^ kit (Insphero Inc., Wagistrase, Schlieren, Switzerland) according to the manufacturer’s instruction. Briefly, 400 cells were added to a well of GravityPLUS^TM^ handing drop plate with a complete medium. Then, the microspheres were carefully transferred into the GravityTRAP^TM^ plate after incubation for 3 days. The pictures of microspheres were taken using an inverted microscope (IX73, Olympus, Tokyo, Japan) every other day and the diameter of a microsphere was determined by measuring two mutually perpendiculars (length and width) in a plane.

### Generation of paclitaxel-resistant cell line OV3R-PTX

The human EOC cell line OVCAR-3 was obtained from the American Type Culture Collection (ATCC, Manassas, VA, USA). PTX was purchased from Sichuan Taiji Pharmaceutical Co., Ltd. (Chengdu, Sichuan, China). The PTX-resistant variant of EOC cells was derived from parental OVCAR-3 cells by treating cells with the PTX regimen using a gradually increasing concentration approach (from 0.01, 0.1, 0.5 to 5 μM). Briefly, PTX-sensitive OVCAR-3 cells were plated into a T25 Flask at the cell density of 30% and cultured in RPMI-1640 medium without antibiotics for 24 h. After washing with phosphate-buffered saline (PBS) to remove the cell debris, cells were incubated in complete medium with 0.01 μM PTX for 24 h. Then, PTX medium was replaced with the fresh complete medium without PTX and cells were recovered for another 3-5 days. These cells were again treated with PTX for another 24 h, followed by recovering for several days. The treatment cycle was repeated until cells reached a density of 90%. After washing to remove the dead cells, the survival cells were passaged to another fresh flask, cultured, and treated with PTX again until cells were stable as showing a regular phenotype or morphology. Cells exposed to PTX were passaged 3 times at 0.01 μM, 1 time at 0.1 μM, 12 times at 0.5 μM, 2 times at 1 μM, and 10 times at 5 μM. The duration of cell culture lasted for more than 5 months and the novel cell line was named OV3R-PTX. These cells were then aliquoted and stored in -80°C freezer or liquid-nitrogen tank for further use. For retaining resistant features, cells were re-treated with PTX by adding 0.5 μM PTX into the media at regular intervals after thaw. In subsequent experiments, cells were cultured in the absence of PTX for at least one passage.

### Measurement of the half-maximal inhibitory concentration (IC_50_) and drug resistance index (DRI)

The cell viability was detected using the WST-1 assay (Roche Diagnostics, Indianapolis, IN, USA) and cell resistance to PTX was calculated by the IC_50_ and DRI. Briefly, OVCAR-3 and OV3R-PTX cells were respectively incubated in 96-well plates at 5 × 10^3^ cells/well for 24 h. These cells were then treated with different concentrations of PTX (0, 0.0001, 0.001, 0.01, 0.1, 0.5, 1, 5, and 10 μM/well) for 48 h. After 10 μL WST-1 reagent was added into each well, the signal in optical density (OD) was detected by a microplate reader (BioTek Epoch, Winooski, VT, USA) at the absorbance of 450 nm wavelength. IC_50_ was calculated using GraphPad Prism 5 (GraphPad Software Inc., San Diego, CA, USA). DRI was calculated according to the following equation, DRI = IC_50_ of drug-resistant cells / IC_50_ of drug-sensitive cells. The IC_50_ and DRI were measured again after 1 and 6 months of storage, respectively, to determine the stability of cell resistance to PTX.

### Tumor xenograft mouse model

The animal experiment was approved by the Ethics Committee of Jinshan Hospital, Fudan University. Female 8-week-old BALB/c nude mice (Shanghai Super-B&K Laboratory Animal Corp. Ltd., Shanghai, China) were labeled and divided into two groups randomly: PTX-sensitive OVCAR-3 and PTX-resistant OV3R-PTX (each n = 10). Each mouse was subcutaneously injected with 5 × 10^6^ cells/150 μl RPMI-1640 without FBS into the right flank of a mouse. Body-weight and tumor volume (Volume = tumor length × width^2^ / 2) were measured every 2 days. Animals were sacrificed on day 21 and whole tumors were excised and photographed.

### Fluorescence-activated cell sorting (FACS)

A single-cell clone was obtained from the OV3R-PTX population using a FACS technique. Briefly, cells were plated in a T75 Flask and cultured. After the cell confluency reached to 80%, the cells were treated with 10 μg/ml of fluorescent glycan nanoparticle for 4 h. After washing twice with ice-cold PBS (pH 7.4), the cells were trypsinized, centrifuged, and resuspended in PBS supplemented with 2% FBS at a density of 1 × 10^5^ cells/ml. The cell population was immediately sorted using flow cytometry (BD FACSAria II, BD Biosciences, USA). A single cell was selected and re-cultured to generate a monoclonal cell line which was named OV3R-PTX-B4 after the validation of resistance to PTX.

### Spheroid formation of cancer stem cell

Cells were plated in a 6-well ultra-low attachment culture plate (Corning Incorporated, Corning, NY, USA) at a density of 1 × 10^3^ cells/well and cultured in serum-free DMEM/F12 cell medium (Gibco-Invitrogen) supplemented with 20 ng/ml epidermal growth factor (EGF, ThermoFisher, Waltham, MA, USA), 20 ng/ml basic fibroblast growth factor (bFGF, ThermoFisher), 4 μg/ml heparin (Sigma-Aldrich), and 0.4 μg/ml B27 (ThermoFishher) and chased every 4 days until day 11. The growth characteristics of spheroids were observed and the diameter of a micro-spheroid was determined as indicated above.

### Cell transfection of siRNA and plasmid

The STAT1-overexpressing plasmid was generated in this laboratory as described in our previous report. [17] STAT1-siRNAs were synthesized from GenePharma (Shanghai, China) and the sequences were listed in Supplementary Table 1. Briefly, after seeding in 6-well plates for 24 h, cells were transfected with 5 nM siRNA or 2.5 μg STAT1 plasmid for 6 h. After removing transfection reagents, cells were further incubated for 48 h. The transfected cells were lysed for mRNA and protein extraction or further used for sphere or colony formation assays.

### Flow cytometry analysis

After seeding in a 6-well plate at a density of 2 × 10^5^ cells/well and incubating for 48 h, cells were detached by trypsin-EDTA and centrifuged. For NANOG and OCT4 analysis, cells were incubated with 0.1% Triton X-100 plus 0.1% FBS in PBS for 40 min and then washed with PBS three times. After adding fluorochrome-conjugated antibodies (Becton, Dickinson and Company, NJ, USA), the cells were incubated for 1 h at room temperature. After washing, cells were resuspended with PBS containing 1% FBS and the cell population was immediately analyzed by Gallios Flow Cytometer (Beckman Coulter, Calif, USA).

### Western blot analysis

Cells were lysed using sodium dodecyl sulfate (SDS) Lysis Buffer (KeyGEN BioTECH, Nanjing, China) supplemented with 1% PMSF and phosphatase inhibitors (KeyGEN BioTECH). Equal amounts of protein were separated on 8% SDS-PAGE and transferred to a polyvinylidene fluoride membrane (Millipore, Billerica, MA, USA). The membrane was then incubated with a primary antibody at 4°C overnight, followed by incubation with horseradish peroxidase-conjugated goat anti-rabbit or anti-mouse IgG (Cell Signaling Technology, Inc., Danvers, MA, USA) for 1 h at room temperature. Rabbit monoclonal anti-STAT1, anti-CD133, anti-OCT4, anti-β-tubulin, and mouse monoclonal anti-CD44 and anti-NANOG antibodies were purchased from Cell Signaling Technology, Inc., Danvers, MA, USA. Anti-β-actin and anti-GAPDH antibodies were from Proteintech Group, Inc., Rosemont, USA. The signals were detected using Immobilon™ Western Chemiluminescent HRP Substrate (Millipore) and quantified using the Tanon-4500 Gel Imaging System with GIS ID Analysis Software v4.1.5 (Tanon Science and Technology Co., Ltd., Shanghai, China).

### Quantitative real-time polymerase chain reaction (qRT-PCR)

Total RNA was extracted from cells using the RNA-Quick Purification kit (ES Science, Shanghai, China). RNA sample (500 ng) was reverse-transcribed using a Transcriptor First Strand cDNA Synthesis Kit (Roche Diagnostics, Indianapolis, IN, USA). The primers of STAT1 and GAPDH were synthesized (GENEWIZ, Suzhou, China). The sequences of primers were listed in Supplementary Table 1. PCR amplification was performed on the 7300 Real-Time PCR system (Applied Biosystems, Foster City, CA, USA) using FastStart Universal SYBR Green Master reagent (Roche Diagnostics).

### Bisulfite modification of DNA and methylation-specific polymerase chain reaction (MSP)

The CpG-rich promoter region of STAT1 was predicted using the UCSC Genome Browser database (http://genome.ucsc.edu/) [46]. For MSP and DNA sequencing, the full-length CpG-rich promoter region (834 bp) was divided into 3 fragments (A, B, and C) based on the design of the primers synthesized by GENEWIZ (Supplementary Table 1). After extracting genomic DNA from OVCAR3 and OV3R-PTX cells using the TIANamp Genomic DNA Kit (TIANGEN BIOTECH, Beijing, China), bisulfite conversion of unmethylated cytosine to uracil was performed using the EZ DNA Methylation-Gold kit (ZYMO RESEARCH, Irvine, CA, U.S.A). The bisulfite-modified DNA was then used as a template for MSP using the Methylation-Specific PCR Kit (TIANGEN BIOTECH), followed by Sanger sequencing.

### Demethylation treatment

OV3R-PTX cells were treated with 0, 10, and 100 μM demethylating agent 5-Aza-2’-deoxycytidine (5-Aza) (Sigma-Aldrich, St Louis, MO, USA) for 3 days. Dimethyl sulphoxide (DMSO) was used as solvent control. After treatment, cells were harvested for RNA and protein extraction.

### CCK-8 viability assay

OV3R-PTX-B4 cell viability was measured using a Cell Counting Kit-8 (CCK-8; Dojindo Laboratories, Kumamoto, Japan). Briefly, cells were plated in a 96-well culture plate at a density of 3 × 10^3^ cells/well and cultured for 24 h. After transfection with STAT1 plasmid for 6 h, cells were further incubated for 24, 48, 72, and 96 h. After 10 μl CCK-8 reagent was added to each well, the signal in OD was read by a microplate reader (BioTek Epoch) at 450 nm. The experiment was repeated at least three times. Cell viability was calculated according to the following equation: Cell Viability (%) = OD value of experiment / OD value of control × 100%.

### Cell colony formation assay

OV3R-PTX-B4 cells were seeded into a 6-well plate at a density of 800 cells/well. After culture for 7 days, small colonies were visible. The culture medium was then changed every two days. After culture for 11 days, the colonies were fixed with 4% paraformaldehyde for 30 min, followed by staining cells with crystal violet (Sigma-Aldrich) for 15 min. After washing, the colonies were photographed under an inverted microscope and the number of colonies was counted.

### Bioinformatics analysis

The data of the overall survival (OS), progression-free survival (PFS), and post-progression survival (PPS) of OC patients with STAT1 mRNA detection were extracted from Kaplan-Meier Plotter (https://kmplot.com/analysis/). Patients were divided into two groups by the median level of STAT1 (low and high expressions). The Kaplan-Meier survival plot, the hazard ratio (HR) with 95% confidence intervals (CIs), and the log-rank P value were calculated in all patients and patients who had PTX treatment, respectively.

### Statistical analysis

Data were analyzed by GraphPad Prism 5 (GraphPad Software Inc., San Diego, CA, USA). A Student’s *t*-test was applied for comparison between the two groups. Results were presented as the mean ± the standard error of the mean (SEM). A P-value of less than 0.05 was considered a statistically significant difference between the two groups.

## Supplementary Material

Supplementary Figures

Supplementary Table 1
