# Supplementary material for: Highly expressed STAT1 contributes to the suppression of stemness properties in human paclitaxel-resistant ovarian cancer cells
Source: Aging (Albany NY). 2020 Jun 9;12(11):11042–60. doi: 10.18632/aging.103317 (PMC7346083; doi:10.18632/aging.103317)
Supplement: Supplementary Figures [file aging-12-103317-s002..pdf]

SUPPLEMENTARY FIGURES

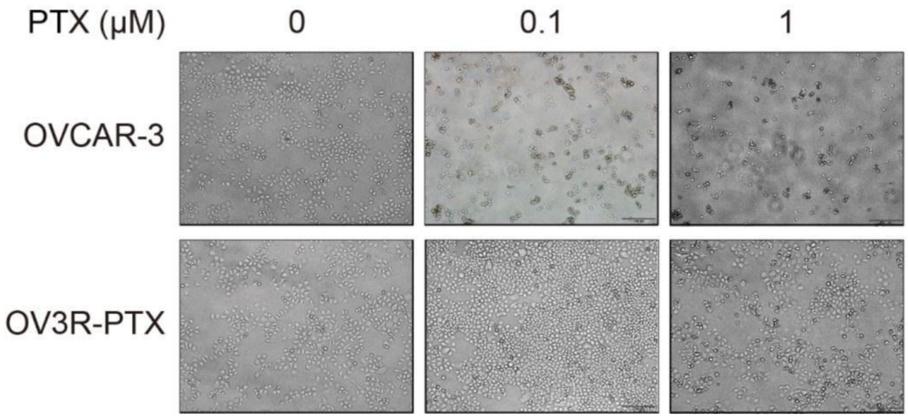

Supplementary Figure 1. Establishment of the paclitaxel-resistant cell line. OVCAR-3 and OV3R-PTX cells were treated with 0, 0.1, and 1 μM PTX for 48 h. OVCAR-3 cells were sensitive to PTX, whereas OV3R-PTX cells were resistant to PTX. The pictures of cell growth were taken by phase-contrast microscopy. Representative images are shown. Original magnification, × 100; scale bar, 200 μm.

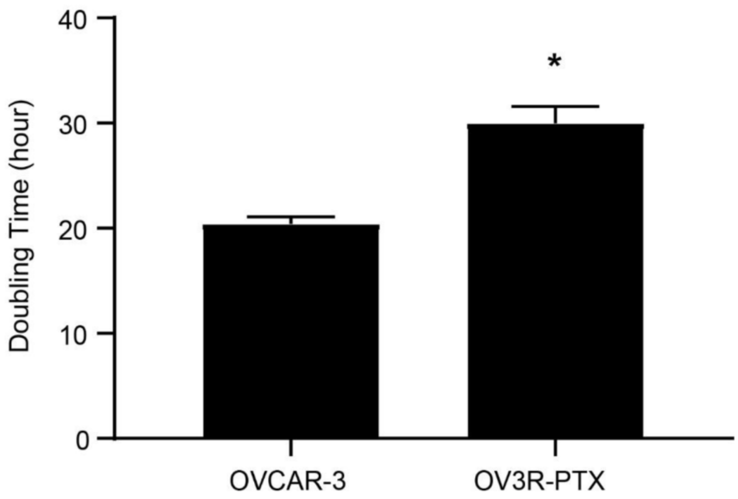

Supplementary Figure 2. Doubling time of cell growth. PTX-sensitive OVCAR-3 and PTX-resistant OV3R-PTX cells were cultured in monolayer 2D culture. OV3R-PTX cells had longer doubling time compared with OVCAR-3 cells. Data are presented as mean ± SEM. n = 3 independent experiments; *, $P < 0.05$ compared to OVCAR-3 cells.

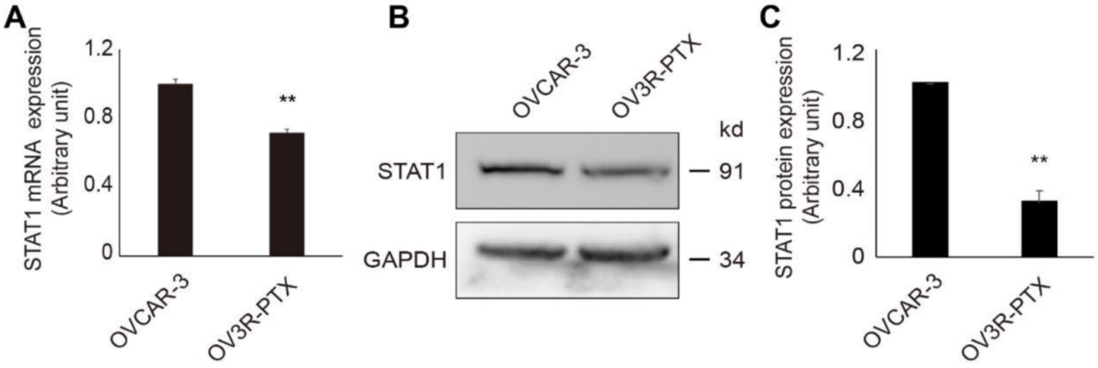

Supplementary Figure 3. Expression of STAT1 in OVCAR-3 and OV3R-PTX cells. (**A**) STAT1 mRNA expression detected by qRT-PCR. (**B**) STAT1 protein expression detected by Western blot. GAPDH was used as a loading control. Representative images of blotting are shown. (**C**) Semi-quantitative analysis of the relative optical density of protein bands in **B**. The expression of STAT1 was significantly lower in OV3R-PTX cells than OVCAR-3 cells. n = 3; **, $P < 0.01$.

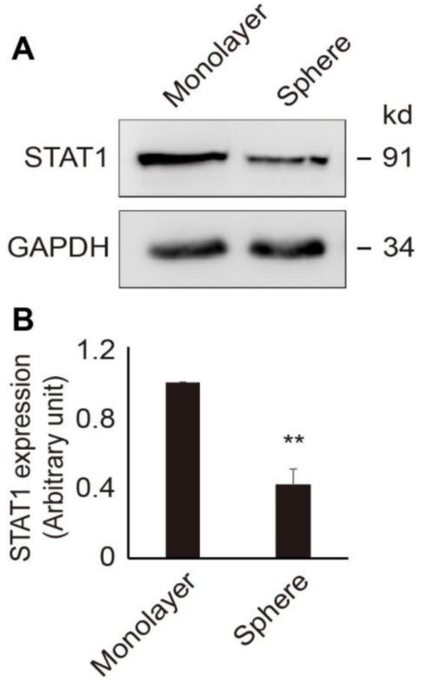

Supplementary Figure 4. Expression of STAT1 in OV3R-PTX-B4 cells. (**A**) STAT1 protein expression detected by Western blot. GAPDH was used as a loading control. Representative images of blotting are shown. (**B**) Semi-quantitative analysis of the relative optical density of protein bands in **A**. The expression of STAT1 was significantly lower in OV3R-PTX cells than OVCAR-3 cells. n = 3; **, $P < 0.01$.
