# Supplementary material for: Highly expressed STAT1 contributes to the suppression of stemness properties in human paclitaxel-resistant ovarian cancer cells
Source: Aging (Albany NY). 2020 Jun 9;12(11):11042–60. doi: 10.18632/aging.103317 (PMC7346083; doi:10.18632/aging.103317)
Supplement: Supplementary Table 1 [file aging-12-103317-s001..pdf]

SUPPLEMENTARY TABLE

Supplementary Table 1. Primer and siRNA sequences used in experiments.

Target primer and siRNA	Sequence 5' → 3'	Position in sequence
**qRT-PCR primers**		
STAT1		
Forward	TCCGTTTTCATGACCTCCTG	nt 10179-10198
Reverse	TGAATATTCCCCGACTGAGC	nt 18088-18107
GAPDH		
Forward	GCACCGTCAAGGCTGAGAAC	nt 249 to 268
Reverse	TGGTGAAGACGCCAGTGGA	nt 368 to 386
**siRNA**		
STAT1-siRNA		
Sense	GCGUAAUCUUCAGGAUAAUtt	nt 649 to 667
Antisense	AUUAUCCUGAAGAUUACGCtt	
Control-siRNA		
Sense	UUCUCCGAACGUGUCACGUtt	Scramble
Antisense	ACGUGACACGUUCGGAGAAtt	
**MSP and Sanger sequencing primers**		
Fragment A		
Forward	GAGGTAGGAAAAAGTAAGAAGG	nt 4542-4563
Reverse	CTAAATAAACTACAACCCAATCC	nt 4874-4896
Fragment B		
Forward	GGATTGGGTTGTAGTTTATTTAG	nt 4874-4896
Reverse	CAACCAAACCCCTCCACAAACTC	nt 5098-5120
Fragment C		
Forward	GAGTTTGTGGAGGGGTTTGGTTG	nt 5098-5120
Reverse	CTCAAAACACATTCTTCCTC	nt 5356-5375

The sequence position of STAT1 and GAPDH primers is indicated based on the sequences in the GenBank with accession # NG_008294.1 and # NM_002046, respectively. MSP, methylation-specific polymerase chain reaction; nt, nucleotide.
